# Associations between hyperacusis and psychosocial work factors in the general population

**DOI:** 10.1007/s00420-018-1356-x

**Published:** 2018-09-07

**Authors:** Johan Paulin, Maria Nordin, Maj-Helen Nyback, Steven Nordin

**Affiliations:** 1 Department of Psychology, Umeå University, 901 87 Umeå, Sweden; 2 YH Novia/Novia University of Applied Sciences, Vaasa, Finland

**Keywords:** Psychosocial work environment, Effort–reward imbalance, Worry at work, Social support, Emotional support

## Abstract

**Purpose:**

We investigated the association between hyperacusis and aspects of psychosocial work environment in a general population. The objectives were to investigate (1) prevalence and characteristics (among age, sex, access to social support at home, education, smoking, physical exercise, and perceived general health) of hyperacusis in a general working population and (2) associations between hyperacusis and psychosocial factors in the work environment. The psychosocial work aspects included effort, reward, overcommitment, worry, and social and emotional support.

**Methods:**

Using data from a sample stratified for age and sex from the Österbotten Environmental Health Study in Finland, currently employed participants with self-reported hyperacusis and referents were compared on questionnaire instruments quantifying six aspects of their psychosocial work environment.

**Results:**

Among 856 currently employed participants, 47 constituted a hyperacusis group and 809 a reference group. The hyperacusis group scored significantly higher than the referents on worry at work, social support at work, and reward at work, but not on emotional support at work, work overcommitment, or effort at work. About 40% of the hyperacusis group scored on the upper quartile of the three former work environment factors, with odds ratios ranging from 1.91 to 2.56.

**Conclusions:**

The results suggest that worrying about aspects at work, perceiving low social support, and not perceiving being rewarded at work are associated with hyperacusis.

## Introduction

Hyperacusis is estimated to afflict 8–9% of the general population (Andersson et al. 2002; Fabijanska et al. 1999; Paulin et al. 2016), and is characterized by negative reactions to sounds at lower levels than to which the majority reacts. Symptoms include disturbed sleep, fatigue, negative emotional well-being, anxiety, and concentration difficulties (Jüris et al. 2013; Pienkowski et al. 2014), which may lead to sick leave.

Hypersensitivity to sounds may have an effect on working life. Working in a noisy environment can have various health outcomes depending on type of exposure. A work environment with loud and unexpected bursts of sound, such as an industrial setting or a mine, may cause hearing damage, resulting in deafness, tinnitus, and possibly also hyperacusis. However, sounds do not have to be loud to be adverse. Performance on cognitively challenging tasks is negatively affected by auditory distractors at irregular intervals, independently of its loudness (Beaman 2005; Geisser et al. 2008; Ljungberg et al. 2002). In other words, the stressor is not necessarily the loudness of the stimulus, but rather the level of predictability and perceived control over the stimulus.

Lack of control along with noise is two well-known stressors. Thus, it is likely that persons with hyperacusis also suffer from stress (Nordin et al. 2013a). Another well-known stressor is work, and multiple theories on the relationship between work stress and health have been presented (Bakker and Demerouti 2007; Karasek and Theorell 1992; Siegrist 1996).

It is well known that psychosocial factors contribute to stress at the work place. High effort along with low monetary and confirmative rewards at work have been shown to increase the risk of, for example, cardiovascular disease (Dragano et al. 2017), whereas good relationships, atmosphere, and support from work decrease the risk of ill-health in the form of, for example, stress and sleep disturbance (Luchman and González-Morales 2013; Nordin et al. 2012). Individual factors are important to consider as well, since psychosocial factors arise in the interaction between organizational and personal demands and resources (Bolin 2009). Joksimovic et al. (2002) found increased risk of musculoskeletal pain among employees who were high on work overcommitment or found it difficult to let go of work during leisure time. There are also indications of associations between effort–reward imbalance and musculoskeletal pain (Koch et al. 2014). Thus, stress due to psychosocial and individual factors can contribute to several types of symptoms, and hyperacusis may well be one of them. Since stress causes vigilance and alerted attention (Liston et al. 2009), it is not unlikely that persons who perceive stress also may respond with alertness to sound. The other way around, thus sensitivity to sound increasing sensitivity to stress, is also be possibly, since unwanted sounds by definition are classified as noise, and noise is a strong stressor (Carter and Beh 1989; Mosskov and Ettema 1977).

The previous research show that women who suffer from high emotional exhaustion and exposure to acute stress have 2–3 times increased risk of developing hyperacusis compared to women with low emotional exhaustion (Hasson et al. 2013). Noise has also been shown to enhance stress and impair cognitive performance (Fried et al. 2002; Tafalla and Evans 1997). However, to our knowledge, there is no documentation on the association between psychosocial work factors and hyperacusis, but prior studies do indeed imply that stress plays an import role important also in hyperacusis.

Given this background, the objectives of the study were, by means of data from the Österbotten Environmental Health Study, to investigate (1) prevalence and characteristics (among age, sex, access to social support at home, education, smoking, physical exercise, and perceived general health) of hyperacusis in a general working population and (2) associations between hyperacusis and psychosocial factors in the work environment. The psychosocial work aspects included effort, reward, overcommitment, worry, and social and emotional support.

## Methods

### Study population and sample

The Österbotten Environmental Health Study is a Finnish questionnaire survey with the overall aim of obtaining better understanding for environmental hypersensitivity, asthma, and allergy, with special interest in the working population. A representative sample from the county of Österbotten was randomly selected from the population registry after stratification for sex and the age strata 18–29, 30–39, 40–49, 50–59, 60–69, and 70–79 years. The sample size was based on the lowest expected prevalence for a specific environmental intolerance by sex, which was symptoms attributed to electromagnetic fields for men (1.1%; Hillert et al. 2002). Precision was set to 0.55%, and with a level of confidence of 95%, the sample size was calculated to 1382 men according to Daniel (1999). Since the sex distribution in Österbotten was nearly equally distributed, the number of women needed was considered the same as for men (*n* = 1382). With an expected response rate of 60%, the sample size was estimated to 4607 participants. The questionnaire was sent by mail to 4607 adults (aged 18–79 years), of which 33.3% (*n* = 1535) agreed to participate. The age and sex distribution of these 1535 participants is given in Table 1. The average retirement age in Finland at the time of the survey (2012) was 60 years (Finnish Centre for Pensions 2018). In total, 3232 persons in the age of 18–59 years were invited, of which 930 agreed to participate. This resulted in a response rate of 28.8% for the sample at working age.

**Table 1:** Numbers of respondents (and percentage of those invited) across age and sex strata in the Österbotten Environmental Health Study

Age (years)	Women	Men
18–29	128 (28.6)	70 (14.2)
30–39	121 (36.0)	80 (21.3)
40–49	140 (37.4)	80 (19.7)
50–59	192 (46.0)	123 (29.5)
60–69	186 (44.2)	169 (39.5)
70–79	131 (44.9)	115 (43.5)
Total sample	898 (39.7)	637 (27.2)

The present study used a subsample of the entire sample, focusing on hyperacusis and the psychosocial work environment. Due to the focuses on the work environment, an inclusion criterion was having an occupation. Thus, individuals were included if they were an employee/employer (including participants who were long-term sick listed, on leave of absence or on parental leave). This resulted in a total sample of 856 participants. Forty-seven of those made up the hyperacusis group, since they responded affirmatively to the question “Do you have a hard time tolerating everyday sounds that you believe most other people can tolerate?”. The remaining 809 participants constituted a reference group. Two participants in the hyperacusis group and 39 in the referent group were identified as long-term sick listed, on leave of absence or on parental leave.

### Procedure

A questionnaire was sent to the participants with the instruction to return it via mail with prepaid postage. Those who did not respond to the first invitation received up to two reminders. The sample responded during the period March–April, 2012. Informed consent was obtained from all participants by informing them in a cover letter that by responding to and returning the questionnaire that they were giving their informed consent.

### Questions and questionnaire instruments

The questionnaire included questions about demographics, lifestyle, and perceived general health as well as several questionnaire instruments. The 11-item Noise Sensitivity Scale (Nordin et al. 2013b) was used to quantify affective reactions to and behavioral disruptions by environmental sounds. The scale consists of 11 statements (e.g., “At movies, whispering and crinkling candy wrappers disturb me”) for the individual to respond to on a Likert scale. The scale is unidimensional and has good reliability and validity as well as normative data (Nordin et al. 2013b). High score represents high level of reactions and disruptions.

A 10-item version of the Effort–Reward Imbalance instrument (Siegrist et al. 2009) with its two subscales, effort and reward, was used to assess demands and rewards at work. Three items measure effort (e.g., “I have constant time pressure due to a heavy work load”), and seven items measure reward (e.g., “I receive the respect I deserve from my colleagues”). High scores represent high effort and lack of reward. Cronbach’s *α* for this study was 0.80 for the effort subscale and 0.84 for the reward subscale.

Overcommitment at work was assessed with the 6-item Work Overcommitment Scale (Siegrist et al. 2004). It measures the tendency to worry about and fixate on work-related tasks also when not at work. The instrument concerns thoughts about extent to which work takes up time and resources (e.g., “I start thinking of work immediately when I wake up”). High score represents high overcommitment. Cronbach’s α for this study was 0.51.

Worry at work (Peter et al. 1998) is a 10-item instrument measuring extent to which worry is caused at the workplace (e.g., “To have an accident at work”, “relocation of job”, and “job reorganization”). High score represents high worry. Cronbach’s α for this study was 0.70.

Social support at work was assessed with the instrument Demand–Control–Support, which measures general atmosphere and mood at the workplace with regard to both colleagues and superiors (Johnson and Hall 1988; Karasek and Theorell 1992). The instrument features seven items (e.g., “There’s a calm and pleasant atmosphere at my work”). High score represents lack of social support. Cronbach’s *α* for this study was 0.86.

Emotional support at work was quantified the 3-item version of the instrument Availability of Attachment (Nordin 2006). It measures the availability of close, affectionate relationships, and social support (e.g., “There is a specific person from whom I feel I really can get support”; Henderson et al. 1980). High score represents lack of emotional support. Cronbach’s *α* for this study was 0.75.

### Statistical analysis

Missing values on the questionnaire instruments for the variables worry at (12.6%), social support at work (10.3%), emotional support at work (10.3%), reward at work (8.9%), effort at work (7.6%), and work over commitment (8.7%) were estimated with multiple imputations using fully conditional Markov chain Monte Carlo methods with 10 maximum iterations by means of which five imputed data sets were created. The estimated values were obtained by pooling the five data sets.

Regarding the characteristics of hyperacusis, the hyperacusis and referent groups were compared on various variables with independent *t* test and Chi-square analyses. The participants were categorized as either high (upper quartile; meeting criteria for poor psychosocial work environment) or low (remaining quartiles) on the work-related factors. Logistic regression analyses were conducted to obtain crude and adjusted (for sex, which differed between the hyperacusis group and referents) odds ratios (ORs) for the various aspects of poor psychosocial work environment for the hyperacusis group. The *α*-level was set at 0.05. The statistical analyses were performed using IBM SPSS Statistics 24 (IBM Corporation, New York).

## Results

In the total sample, 47 (5.5%) reported hyperacusis. This group scored significantly higher (mean = 37.5, SD = 8.29) than the referents (mean = 26.6, SD = 7.45) on affective and behavioral reactions to environmental sounds (11-item Noise Sensitivity Scale). The hyperacusis group typically had had their intolerance for several years (mean = 7.4, SD = 9.2) and two-thirds (66%) of the group experienced symptoms daily or weekly. Compared to the reference group, the hyperacusis group consisted to a significantly larger proportion of women, and reported poorer general health according to *t* test and Chi-square analysis. The groups did not differ significantly regarding age, access to social support at home, physical exercise, education, or smoking. The results are shown in Table 2.

**Table 2:** Description of the hyperacusis and referent groups with respect to demographics, lifestyle, perceived general health and affective and behavioral reactions to sound, and comparisons between the hyperacusis group and referents with *t* test and Chi-square analysis

	Hyperacusis (*n* = 47)	Referents (*n* = 809)	*p* value
Age (years; mean ± SD)	42.23 ± 11.56	45.2 ± 11.89	0.301
Women, *n* (%)	35 (74.5)	482 (59.6)	0.042
Living alone (single household with no adult children living at home), *n* (%)	9 (19.1)	111 (13.7)	0.297
Education (highest level), *n* (%)			0.165
Compulsory school	1 (2.1)	81 (10.0)	
Senior high school	22 (46.8)	372 (46.0)	
College/University	24 (51.1)	343 (42.4)	
No response	0 (0)	13 (1.6)	
Smoking *n* (%)	7 (14.9)	95 (11.7)	0.517
Physical exercise, *n* (%)			0.914
Once a month or less	6 (12.8)	134 (16.6)	
2–4 times/month	15 (31.9)	240 (29.7)	
2–3 times/week	18 (38.3)	301 (37.2)	
More than 3 times a week	8 (17.0)	127 (15.7)	
No response		7 (0.9)	
Perceived general health, *n* (%)			0.005
Very good	14 (29.8)	335 (41.4)	
Good	16 (34.0)	329 (40.7)	
Poor	17 (36.2)	140 (17.3)	
No response		5 (0.6)	

Table 3 describes the hyperacusis and referent groups with respect to the six psychosocial work environment factors. The hyperacusis group had significantly higher scores than the referent group on worry, social support, and reward, whereas the groups did not differ significantly with respect to emotional support, overcommitment or effort.

**Table 3:** Mean ± SD scores on psychosocial work environment factor in the hyperacusis and referent groups, and group comparisons with results from *t* test and Chi-square analysis

	Hyperacusis (*n* = 47)	Referents (*n* = 809)	All (*n* = 856)	*p* value
Worry at work	3.05 ± 2.35	2.06 ± 1.91	2.12 ± 1.95	0.003
Lack of social support at work	14.26 ± 4.24	12.52 ± 3.99	12.62 ± 4.02	0.036
Lack of reward at work	7.94 ± 6.76	7.66 ± 5.72	7.67 ± 5.80	0.024
Lack of emotional support at work	6.34 ± 2.16	5.84 ± 2.05	5.86 ± 2.06	0.105
Work overcommitment	14.36 ± 3.09	14.10 ± 2.98	14.11 ± 2.98	0.084
Effort at work	16.48 ± 2.71	14.61 ± 2.91	14.71 ± 2.90	0.214

**Fig. 1:**
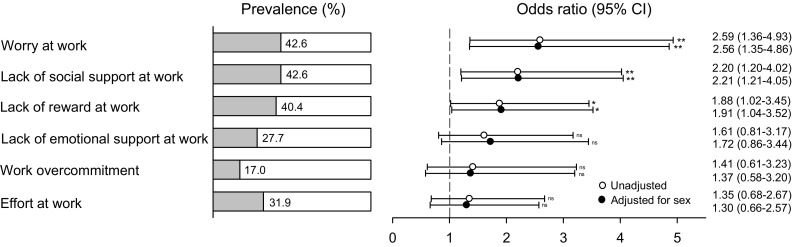
Percentage of employed participants with hyperacusis who also had various aspects of poor psychosocial work environment (upper quartile) as well as odds ratios, confidence intervals (CIs), and *p* values when unadjusted (crude) and adjusted for sex

Figure 1 shows percentage of employed participants with hyperacusis with poor psychosocial work environmental factors, as well as ORs. Between 17.0 and 42.6% of the hyperacusis group scored in the upper quartile of the six work environment factors. Relative to the referent group, there was a significantly increased risk in the hyperacusis group, when adjusted for sex, of scoring high on worry, social support, and reward (ORs = 1.91–2.56), but not on scoring high on emotional support, overcommitment or effort (ORs = 1.30–1.72). The unadjusted ORs were in general very similar to corresponding adjusted ORs.

## Discussion

The aim of the study was to investigate prevalence and characteristics of hyperacusis in a general working population, and associations between hyperacusis and psychosocial work factors. Similar to the previous studies (Jüris et al. 2014; Paulin et al. 2016), our findings show that most of those with hyperacusis were women and that they compared to the referent group reported poorer general health. This is in line with a study of a hyperacusis in the general population, showing higher than normal prevalence of posttraumatic stress disorder, chronic fatigue syndrome, generalized anxiety disorder, depression, exhaustion syndrome, fibromyalgia irritable bowel syndrome, migraine, and tinnitus. Moreover, also similar to the present study, the afflicted individuals had had the intolerance for, on average, 7 years, and two-thirds of the group experienced hyperacusis symptoms either daily or weekly (Paulin et al. 2016). Hence, in general, hyperacusis appears to be a long-term condition that affects afflicted individual on a frequent basis.

Regarding the association between psychosocial work environment and hyperacusis, the results show that worrying about things at work, perceiving low social support at work and not feeling rewarded at work were significantly associated with hyperacusis. Perceiving poor emotional support, being overcommitted and spending much effort at work also increased the risk associated with hyperacusis, although not significantly. Thus, worry, social relations, and being rewarded and confirmed at work seem to be of importance for hyperacusis.

Worry is a prominent stressor. Worrying about work and perceiving lack of support and reward from coworkers and supervisors are likely to contribute to stress. The linkage between stress and hyperacusis is not yet understood, but it has previously been found that persons suffering from hyperacusis also are more likely to experience psychological distress such as general anxiety, depression, or phobia (Aazh and Allott 2016; Auerbach et al. 2014; Jüris et al. 2013; Sahley and Nodar 2001).

Interestingly, lack of emotional support was not found to be associated with hyperacusis in the same way as lack of social support from work in the form of atmosphere among coworkers. The previous results show that emotional support at work is not associated with, for instance, sleep problems, whereas this is the case when being socially integrated at work. However, emotional support from home has been shown to be of importance for sleep (Nordin et al. 2012). The authors reasoned that emotional support is more likely to be received from home, whereas good atmosphere at work is needed to get things done. It is, for example, important that coworkers dare to ask each other for help. If this is not the case, stress may become evident and tangible, since you will have to solve the work problems on your own.

Persons with hyperacusis in the present study were more likely not to perceive rewards at work. Reward can be given in many ways, and the reward scale from the Effort–Reward Imbalance includes both monetary and confirmatory rewards. Not experiencing that you are paid well enough or that your work is not being confirmed by others may add to feelings of distress (Fahlén 2008; Siegrist 1996, 2010).

Whether or not the psychosocial work environment has contributed to the development of hyperacusis cannot be concluded from the results from this cross-sectional study. Environmental noise is a distractor even among healthy adults, increasing the mental workload and reducing task performance by hijacking available cognitive resources (Szalma and Hancock 2011). It may be more difficult than normal in hyperacusis to perform well at work, since sounds are perceived as annoying and stress-inducing (Sandrock et al. 2009), and further indirect support for evoked stress is reported sleep problems in hyperacusis (Nordin and Nordin 2016). Disturbed sleep may be an indicator of both being easily disturbed due to a hypersensitivity and of stress, since stress is incompatible with sleep. Nevertheless, it is not unlikely that people with hyperacusis suffer more from being tired and fatigued which, in turn, makes it more difficult to perform work and maintain good relations with others (Christian and Ellis 2011).

The present study has both strengths and limitations. Strengths include being population-based and stratified for age and sex, having a decent sample size (*n* = 1535), and the county of Österbotten having an age and sex distribution that is very similar to that of Finland in general (Regional Council of Ostrobothnia 2015). One limitation of the study is the low response rate (28.8%) among the working age group, which limits the generalizability of the results to the general working population. The response rate was higher in women than in men, which also may have consequences for the generalizability if women and men respond differently to questions about their psychosocial work environment. However, there is support for the notion that such differences do not clearly exist (Sverke et al. 2016). Another limitation is the small size of the sample with hyperacusis (*n* = 47), resulting in low statistical power. This may have contributed to some of the differences in psychosocial factors not reaching statistical significance. Furthermore, since the focus of the study was on environmental health, it is possible that those with an environmental intolerance, such as hyperacusis, are more likely to participate. On the other hand, it is possible that a “healthy worker” effect to some extent has balanced out that effect. Notably, the present results line up well with the previous cross-sectional studies (Andersson et al. 2002; Paulin et al. 2016) showing differences between hyperacusis and referents regarding sex distribution, but not age and education. The internal consistency of the Worry at Work, Availability of Attachment, Effort–Reward Imbalance, and Atmosphere at Work scales was acceptable to good (Cronbach’s *α* = 0.70–0.86). However, the consistency of the Work Overcommitment index was rather poor (0.51), which calls for caution regarding its reliability.

Due to the cross-sectional nature of this study, questions of causality remain unanswered. Do individuals develop hyperacusis partly because of negative psychosocial work environment, or do they perceive the work environment as more negative due to the symptomatology of hyperacusis? We lack details regarding factors such as type of job, length of employment, the previous job experience etc., but we believe that such factors, at least in part, may account for the variance.

To our knowledge, this is the first study to investigate the psychosocial work environment from the perspective of hyperacusis. The results suggest an association between the psychosocial work environment and hyperacusis, with about twofold increased risk of worrying at work and having lack of both social support and reward at work. These relationships, together with a hyperacusis prevalence in the general working population of 5.5%, suggest that a large range of work environments, not only those historically labeled as “noisy”, should be of interest from a hyperacusis perspective. However, further research is needed to more accurately describe the factors relating negative psychosocial work environment to hyperacusis.
